# Six Practical Strategies to Mentor and Sponsor Women in Academic Medicine

**DOI:** 10.2196/47799

**Published:** 2023-05-25

**Authors:** Julie K Silver

**Affiliations:** 1 Spaulding Rehabilitation Hospital Charlestown, MA United States; 2 Department of Physical Medicine and Rehabilitation Harvard Medical School Boston, MA United States; 3 Massachusetts General Hospital Boston, MA United States; 4 Brigham and Women’s Hospital Boston, MA United States

**Keywords:** diversity, women, women in medicine, mentorship, sponsorship, academic medicine

## Abstract

This article focuses on the importance of mentorship and sponsorship for women in academic medicine, including trainees and faculty, and emphasizes the need for flexible and expanded definitions. Both the benefits and potential harms associated with sponsorship are described. There are 6 actionable strategies illustrated that may be added to a multidimensional mentoring model in order to better support women in medicine.

## Introduction

Both mentorship and sponsorship contribute to the success of physicians and scientists in academic medicine. Nevertheless, current definitions of these concepts lack clarity and insufficiently capture certain aspects of best practices as they apply to academic medicine. This may lead to negative effects that disproportionately affect women and people from underrepresented groups.

A report titled *The Science of Effective Mentoring in STEMM*, published by the National Academies of Sciences, Engineering, and Medicine (NASEM), defined *mentorship* as a “professional, working alliance in which individuals work together over time to support the personal and professional growth, development, and success of the relational partners through the provision of career and psychosocial support” [1]. *Sponsorship* was recognized as a subcategory of mentorship and defined as when a “mentor publicly acknowledges the achievements of mentees and advocates for mentees.” Both definitions lack nuance and are inadequate, as even the report notes that people often conflate mentoring with advising, role modeling, coaching, and sponsorship. Moreover, if the mentorship definition requires individuals to “work together over time,” then this will not capture the full continuum of mentoring interactions, including brief meetings such as “speed mentoring.” While some may argue that brief interactions are best thought of as “advising,” this may not give enough credit to mentors—particularly women and individuals who identify with minoritized groups, who do the bulk of this type of “invisible work” (a term used to denote work for which faculty receive little or no recognition and that may negatively impact academic rank evaluations and other types of promotion). Since mentorship is typically part of the formal rank promotion criteria, capturing effort in this domain on one’s curriculum vitae (CV) is important—particularly when considering the low proportions of women at professor and chair levels [2] and the glaring absence of Black and Latina women at the professor level [3].

The NASEM report’s emphasis on sponsorship as a public endeavor fails to capture the power of sponsorship in academic medicine, which ideally happens both in the spotlight and behind the scenes. Furthermore, individuals who are receiving sponsorship might not realize that sponsors acted by mentioning their name—leading to a misperception that they are not receiving this coveted support. Conversely, sponsors, including senior-level men, may be doing more sponsorship than they are receiving credit for, leading to the possibility that they will not be recognized as allies.

Another problem with defining sponsorship narrowly as having someone with power and influence mention an individual’s name and advocating for them is that this does not take into consideration how things actually work in academia. For instance, if a vice-president of a company sponsors a junior person and encourages a fast track promotion based on the potential of that individual this may be seen as helpful and appropriate, whereas if a chair advocates for fast tracking a junior faculty member’s rank promotion based on potential rather than documented accomplishments in a formal promotion dossier, this may be viewed by the promotions committee and dean as interfering and perhaps even unethical.

## Customizing Mentorship and Sponsorship for Academic Medicine

Given the aforementioned definitional limitations, a better description of sponsorship in the context of academic medicine might be as follows: Sponsorship is an action or a process in which someone, generally though not exclusively with more influence and power than the person they are sponsoring, facilitates an opportunity for an individual that might lead to career benefits including, but not limited to, accomplishments required for academic rank promotion and that can be documented on a curriculum vitae. Textbox 1 presents a summary of key definitions and terms used in this paper.

**Key terms and definitions. This information is focused on academic medicine but may be applicable to other fields.**

**Sponsorship**

An action or a process in which a sponsor, generally though not exclusively someone with more influence and power than the person they are sponsoring, facilitates an opportunity for an individual that might lead to career benefits, including but not limited to accomplishments required for academic rank promotion that can be documented on a curriculum vitae.

**Sponsorship party**

An event, in person or virtual, in which participants present prepared sponsorship “asks” and the guests actively focus on supporting the asks by using their power and influence.

**Spentorship**

The act of sponsoring someone and then mentoring them through the process to build their confidence and ensure their success.

**Fourth shift**

The disproportionately large amount of mentoring and sponsorship work that women in medicine do, much of which is uncompensated and unrecognized; the other 3 shifts are their current job, responsibilities outside of work (eg, childcare), and gender equity research and advocacy.

**Push-Pull Mentoring Model**

This model focuses on shared responsibility for the mentor-mentee relationship that is mutually, though not necessarily equally, supportive of both individuals’ career goals, with mentees pushing mentors up and facilitating opportunities in their realm of influence, including but not limited to sponsorship, while mentors are simultaneously pulling them up. This approach acknowledges a shortage of experienced mentors, a lack of protected time for mentorship, and the disproportionate effort that women and people from underrepresented groups spend on mentoring, which in turn may slow or stall their own career progress and contribute to symptoms of burnout.

**Workforce equity triple aim**

The process of simultaneously improving outcomes in 3 key domains: pay equity, promotion equity, and workplace psychological safety and wellness.

Of course, an expanded definition does not address all problems associated with sponsorship. For instance, sponsorship is touted as wholly positive and potential harms are rarely, if ever, considered. Women are often told that they need to find sponsors to be successful, which puts the onus on those who have the least experience and have the weakest connections to find senior people who will advocate for them. Not surprisingly, many women wonder and worry: *How am I supposed to find sponsors?* On the other hand, individuals with the most influence and power (the majority of whom are men) often do not have enough information to figure out how best to engage in sponsorship. This conundrum can lead to both parties feeling pressured, burdened, or discouraged. The first strategy below, in the section “Identify Her Sponsorship Goals,” may help, though it is certainly not a comprehensive antidote.

Another concern is that sponsorship frequently encourages people to stretch beyond the boundaries of their current experience, which may increase stress, exacerbate feelings of not belonging, and feed into the myth that people from marginalized groups lack confidence due to “imposter syndrome” [4]. Sponsorship opportunities might also drain the time of women, trainees, early-career physicians and scientists, and people from minoritized groups, who often experience “time poverty.” In general, these issues can be addressed by what I call *spentorship*, which is strategically combining mentorship and sponsorship to guide people through new experiences in a manner that ensures their success, builds their confidence, and avoids wasting their precious time.

Additionally, there are persistent concerns with mentoring that go beyond definitional issues. For example, the enormous amount of mentoring that many women do, especially those with intersectional identities who are committed to supporting the next generations of diverse people, often drains their time, which has many repercussions, such as them not achieving their own career goals. Mentoring may be considered the “fourth shift” that women work—the other 3 shifts being their regular job, a disproportionately high workload at home, and gender equity research and advocacy [5]. The expectation—whether it is externally driven, internalized, or both—that women in academic medicine will devote an inordinately large amount of time to mentoring can result in slowing their promotions and may contribute to symptoms of burnout.

Although mentoring others is often touted as an altruistic enterprise in which the more senior person should not expect anything in return, this expectation may not be an ideal fit for academic medicine, particularly for mid-career women from underrepresented groups, who often do a disproportionate amount of mentoring; this may negatively impact their ability to achieve their own career goals (eg, writing grants and publishing research) and can result in an increase in stress and burnout symptoms. An alternate model is what I call “push-pull mentoring,” in which both mentors and mentees are supportive of each other’s career goals, and mentors who are more senior “pull” up their mentees while the mentees “push” up their mentors by strategically sponsoring them, as well as by “mentoring up” [6]. In my definition of sponsorship, I intentionally indicated that a sponsor does not have to be someone who has more influence and power than the individual being sponsored. For instance, a trainee or early-career faculty member can push her mentor up now (and for many years to come) by

- Nominating her for a recognition award
- Inviting her to be a coauthor on a paper
- Including her on a grant application
- Mentioning her name as a potential speaker

Sponsorship can come from all directions in academic medicine, and the Push-Pull Mentoring Model focuses on a shared responsibility to intentionally support the mentor and mentee so that both are able to rise.

## Six Actionable Strategies for Mentorship and Sponsorship

Next, I describe 6 practical strategies that demonstrate active mentorship and sponsorship, emphasizing that there is some overlap and recognizing the definitions should allow for flexibility and expansion, particularly since, as I suggest, they are and should be used contemporaneously. This article focuses on gender equity, but of course these strategies may be used to support people from a variety of underrepresented groups. I encourage readers to customize them to fit their own work and the culture of their organizations.

### 1. Identify Her Sponsorship Goals

Schedule time with women individually or in groups and mentor them so that they are able to self-identify 3 to 5 specific sponsorship “asks,” with the goal of creating a sponsorship slide that can be used as part of a presentation and with some of the other exercises described in this article.

Women often hear that they are “undersponsored,” but it can be challenging for people with power and influence to know what would be helpful. Sponsorship asks should avoid being too general (“I want to give more lectures”) or so specific that it would be challenging to find a sponsor.

The following are examples of reasonable asks based on career stage. Resident physician: “I just completed a study focused on how to increase vaccination rates among minoritized groups, and I would like opportunities to give a short lecture on our findings.” Early-career physician: “I have given grand rounds within our institution and regionally on the topic of dermatologic conditions in immunocompromised individuals, and I would like to be invited to give similar lectures at academic medical centers in other states.” Mid-career physician: “I have given traditional grand rounds across the country on [name the topics], and I am ready for the more prestigious invitations that women often do not receive, such as keynotes, lectureships, and visiting professorships.

Ideally, at the end of this exercise, each person creates a “sponsorship slide” with the following information: name, degree, and current position; a professional headshot; 2 to 3 bullet points with biographical information; 2 to 3 bullet points with sponsorship asks; and email address.

### 2: Host a Sponsorship Party

A sponsorship party is a fun event, and it can be held in person or virtually. The guests are people who have power and influence and want to support the participants. The participants present their prepared sponsorship slide and have 2 minutes to introduce themselves and share their asks. Following each participant’s presentation, 3 minutes are allotted for the guests to respond via email to their asks. All guests are encouraged to email every participant during the allotted time, even if it is just to write an encouraging message. If there are 30 guests, then each participant gets 30 emails—some of them offer sponsorship and others are encouraging and build their confidence.

### 3: Facilitate a Round-Robin Sponsorship Announcement

There are many settings where a “mini” sponsorship party or intervention can take place (eg, department meeting). Remind everyone that the primary goal is for people to use their power, influence, and connections to assist in fulfilling an ask. However, advice and words of encouragement are welcome. Consider what parameters are appropriate in a given setting. For example, whether people should ask for things that cost money—asking a group of philanthropists to sponsor a research study is likely appropriate, but asking peers or colleagues to donate money to a GoFundMe campaign might not be.

### 4: Elevate Her to a Bigger Stage

A powerful way to sponsor women in academic medicine is to invite them to speak on stages that they may not normally have access to. For example, a more senior physician who is giving grand rounds could invite a resident physician to give a 10-minute segment of this lecture. I have done this many times, often with me in person and the individual I am sponsoring speaking virtually. A few tips for sponsors to ensure success:

- Get approval from the organizers
- Mentor the individual through the process
- Tell the audience that you are sponsoring her
- Give her an appropriate amount of time (eg, 15 minutes if the audience is expecting to mostly hear from you as the invited speaker)

There are many ways to elevate a mid-career or senior woman to big stages. For example, sponsor her for a lectureship, keynote, or visiting professor role.

### 5: Highlight Her Work

With her permission, write an email about a woman you want to sponsor to influential people whom she has some connection with. The email should explain that you are reaching out on behalf of a colleague to share information about her work. If there is an upcoming “hook” that supports women, then use that to garner interest (eg, “Dear [name], I am writing to you in advance of Women in Medicine Month to see if you might be interested in publishing an article about my accomplished colleague [name], who is an alum of your institution”). I often run this as a 2-hour group workshop and write an email template to save everyone time. I ask each participant to prepare in advance an email that they can forward during the workshop to their assigned partner. This email should contain an updated professional biography, headshot, and list of people they want contacted. Examples of people to contact include editors or communication directors (eg, alums, current medical school publications, hometown newspapers, hospital or medical society newsletters) and institutional leaders (eg, alums or current deans, including those involved with faculty development and diversity, equity, and inclusion).

### 6: Co-author a Letter to the Editor

Co-authoring a letter to the editor about a recent peer-reviewed paper is a great way to sponsor women who could benefit from more experience with the publishing process. This teaches them to think critically, adhere to author guidelines, weigh in on science, and learn to navigate journal submission platforms. If the letter is accepted, then they can document the publication on their CV.

## Conclusion

Each of the 6 strategies can be stand-alone, and there is a synergistic effect when multiple strategies are implemented. They should be incorporated into existing mentoring models so that they are additive. The NASEM mentoring report states that evidence-based mentoring involves a multidimensional model that includes, but is not limited to, the classic dyad mentor-mentee relationship over a period of years [1]. From a workforce perspective, the goals of mentorship and sponsorship should align with what I call the “workforce equity triple aim”: (1) pay equity, (2) promotion equity, and (3) psychological safety and wellness. Supporting the triple aim in workforce equity means that we are able to deliver higher-quality patient care to more people and advance science at a faster rate.

## Abbreviations

CV: curriculum vitae
NASEM: National Academies of Sciences, Engineering, and Medicine
